# The Impact of the Reducing Agent on the Cytotoxicity and Selectivity Index of Silver Nanoparticles in Leukemia and Healthy Cells

**DOI:** 10.3390/nano15241858

**Published:** 2025-12-11

**Authors:** Jovani Guadalupe Aguirre-León, Belkis Sulbarán-Rangel, Edsaúl Emilio Pérez-Guerrero, Antonio Topete-Camacho, Trinidad García-Iglesias, Pedro Ernesto Sánchez-Hernández, Moisés Ramos-Solano, Andrea Carolina Machado-Sulbaran

**Affiliations:** 1 Doctorado en Farmacología, Departamento de Fisiología, Centro Universitario de Ciencias de la Salud (CUCS), Universidad de Guadalajara, Sierra Mojada 950, Independencia Oriente, Guadalajara 44340, Jalisco, Mexico; jovani.aguirre1042@alumnos.udg.mx; 2 Instituto de Investigación en Cáncer en la Infancia y Adolescencia (INICIA), Departamento de Clínicas de la Reproducción Humana, Crecimiento y Desarrollo Infantil, Centro Universitario de Ciencias de la Salud (CUCS), Universidad de Guadalajara, Sierra Mojada No. 950, Independencia Oriente, Guadalajara 44340, Jalisco, Mexico; trinidad.giglesias@academicos.udg.mx (T.G.-I.); pedro.shernandez@academicos.udg.mx (P.E.S.-H.); 3 Departamento de Estudios del Agua y la Energía, Centro Universitario de Tonalá, Universidad de Guadalajara, Nuevo Periférico Oriente, 555, Ejido San José, Tateposco, Tonalá 45425, Jalisco, Mexico; belkis.sulbaran@academicos.udg.mx; 4 Instituto de Investigación en Ciencias Biomédicas, Departamento de Biología Molecular, CUCS, Universidad de Guadalajara, Sierra Mojada 950, Independencia Oriente, Guadalajara 44340, Jalisco, Mexico; edsaul.perezg@academicos.udg.mx; 5 Grupo de Física de Coloides y Polímeros e Instituto de Materiales (iMATUS), Universidad de Santiago de Compostela, 15782 Santiago de Compostela, Spain; antonio.topete@academicos.udg.mx; 6 Laboratorio de Investigación en Cáncer e Infecciones, CUCS, Universidad de Guadalajara, Sierra Mojada 950, Independencia Oriente, Guadalajara 44340, Jalisco, Mexico; moises.ramos@academicos.udg.mx

**Keywords:** nanoparticles, reducing agents, glucose, polyvinylpyrrolidone, cytotoxicity, leukemia

## Abstract

Silver nanoparticles (AgNPs) are widely studied in oncological nanomedicine, although concerns persist regarding their toxicity, elimination, and tissue accumulation. The biological properties of AgNPs depend on the synthesis method and the reducing agent used, which may influence cytotoxicity and cellular metabolism. This study aimed to evaluate the effect of the reducing agent on the cytotoxicity of AgNPs in leukemia (JURKAT) cell lines and peripheral blood mononuclear cells (PBMCs). AgNPs were synthesized via chemical reduction using glucose (GLU) or polyvinylpyrrolidone (PVP) as reducing agents. Nanoparticles were characterized by UV-Vis, FTIR, DLS, zeta potential, and TEM. Cell viability was assessed using trypan blue exclusion, and cytotoxicity was determined using the MTT assay. UV-Vis analysis showed distinct surface plasmon resonance profiles, and FTIR confirmed characteristic functional groups on the nanoparticle surface. DLS and zeta potential values indicated colloidal stability, with PVP-AgNPs presenting a more negative surface charge. TEM revealed greater size heterogeneity in GLU-AgNPs. GLU-AgNPs induced lower cytotoxicity and higher cell viability in JURKAT and PBMCs compared to PVP-AgNPs (*p* < 0.05). Leukemia cells were more susceptible to both nanoparticle types than PBMCs, showing a favorable selectivity index for GLU-AgNPs (SI = 2.44). These findings suggest that biocompatible reducing agents may improve the safety profile of AgNPs.

## 1. Introduction

Silver nanoparticles (AgNPs) have emerged as a highly relevant tool in modern nanomedicine due to their unique properties, including strong antimicrobial activity, cellular penetration, and versatility in diagnostic and therapeutic applications [1,2]. Despite these benefits, concerns about their safety and potential toxic effects have prompted extensive research to understand how physicochemical characteristics—such as size, shape, surface charge, and the reducing agent used during synthesis—influence their biocompatibility [3,4].

The synthesis of AgNPs is commonly achieved through chemical reduction methods, using diverse reducing agents such as sodium borohydride (NaBH_4_) and sodium citrate (Na_3_C_6_H_5_O_7_), which are among the most widely used in industrial and laboratory settings. Sodium borohydride enables the rapid reduction of silver ions, resulting in small and well-defined nanoparticles, whereas sodium citrate serves as both a reducing and stabilizing agent, a technique commonly employed in the classic Turkevich method adapted for AgNP synthesis [5,6]. Although these conventional methods are highly effective and reproducible, they often involve toxic or environmentally harmful chemicals, raising concerns about the biocompatibility and ecological impact of the resulting nanoparticles [7,8].

As an alternative, green synthesis approaches have emerged, utilizing natural, biocompatible reducing and capping agents such as plant extracts, polysaccharides, and simple sugars like glucose (GLU). Glucose is particularly attractive due to its natural abundance, metabolic compatibility, and low cytotoxicity, making GLU-capped AgNPs promising candidates for biomedical applications. In contrast, polyvinylpyrrolidone (PVP)-capped AgNPs represent one of the most widely used formulations in oncological nanomedicine research owing to their excellent colloidal stability and well-established synthesis protocols [8,9]. However, PVP is a synthetic, non-biodegradable polymer that can persist in biological systems. It has been shown to alter protein corona formation, cellular uptake, and inflammatory responses—factors that may compromise therapeutic efficacy or safety [9,10]. Therefore, directly comparing GLU-AgNPs with PVP-AgNPs allows evaluation of whether a green, metabolizable capping agent can provide comparable or superior biocompatibility and anticancer activity while avoiding the potential drawbacks of synthetic stabilizers.

In medicine, AgNPs have been widely explored for diverse applications, including antimicrobial coatings, wound healing, imaging, and targeted drug delivery [11]. Notably, their potential as anticancer agents has attracted significant research interest due to their ability to induce oxidative stress, mitochondrial dysfunction, DNA damage, and apoptosis in cancer cells while sparing normal cells under controlled conditions [5]. Several studies have demonstrated the efficacy of AgNPs against various types of cancer, including leukemia [10]. Moreover, AgNPs synthesized with multiple reducing agents have been shown to induce cytotoxicity, promote the production of reactive oxygen species (ROS), and induce apoptosis [10,12].

These findings suggest that AgNPs could serve as an adjuvant or alternative therapeutic approach in leukemia treatment, particularly by enhancing the selectivity and efficacy of conventional therapies while reducing systemic toxicity [10]. Ongoing research aims to optimize their targeting capacity, biocompatibility, and delivery mechanisms to harness their full potential in oncology.

The selection of appropriate model cells is crucial for assessing nanoparticle toxicity in both pathological and physiological contexts. The JURKAT cell line, derived from human T lymphocytes, is frequently used as a model for leukemia studies, while peripheral blood mononuclear cells (PBMCs) represent primary immune cells essential for immune responses [12]. Given their morphological similarities and functional relevance, these cells provide a suitable system for evaluating whether the choice of reducing agents influences differential cytotoxic responses between malignant and non-malignant immune cells [12].

Previous studies have demonstrated that differences in nanoparticle surface chemistry can strongly influence their cellular uptake and cytotoxicity profiles [13]. Understanding how synthesis parameters, particularly the nature of the reducing agent, affect the physicochemical characteristics and toxicity of nanoparticles is essential for developing safer and more effective nanostructures [11]. Therefore, this work provides a comparison of AgNPs synthesized with GLU and PVP to evaluate their effects on leukemic and healthy cells. The results may offer insights to guide future studies and the design of nanoparticles to enhance therapeutic efficacy while reducing potential risks.

## 2. Materials and Methods

### 2.1. Materials

The reagents required for nanoparticle synthesis included silver nitrate (HyCel, cat. no. no. 1386, Zapopan, Mexico), molecular-grade glucose (Sigma-Aldrich, cat. no. 50-99-7, St. Louis, MO, USA), gelatin (LABESSA, cat. no. GR2085, Caulfield North, Australia), and polyvinylpyrrolidone (PVP) (Sigma-Aldrich, cat. no.9003-39-8, St. Louis, MO, USA).

### 2.2. Synthesis and Characterization of Silver Nanoparticles (AgNPs)

**Figure 1:**
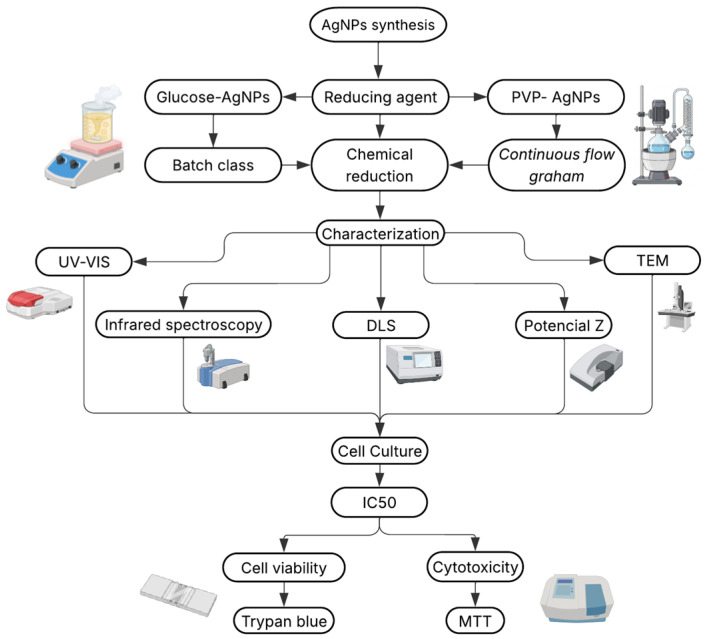
Workflow for the synthesis, characterization, and biological evaluation of AgNPs obtained using two reducing agents: glucose (GLU) and polyvinylpyrrolidone (PVP).

AgNPs were synthesized using two different techniques, each employing a different reducing agent, while maintaining the same overall chemical reduction method. The aim was to compare how the synthesis route—specifically the nature of the reducing agent and the capping agents—influences the physicochemical properties and biological activity of the resulting nanoparticles. A schematic overview of the experimental workflow, including synthesis, characterization, and biological evaluation, is provided in Figure 1. The specific procedures employed in each synthesis method are described below.

#### 2.2.1. GLU-AgNPs

The synthesis of AgNPs using GLU as the reducing agent (GLU-AgNPs) was performed using a previously reported chemical reduction method with minor modifications. Briefly, 100 mL of distilled water was heated to 85 °C, followed by the addition of 0.90 g of molecular-grade GLU and 0.36 g of gelatin. After achieving homogeneity, 0.1 M sodium hydroxide was added dropwise to adjust the pH to 10. Subsequently, 10 mL of 0.1 M silver nitrate solution was added, and the mixture was stirred for 30 min at 85 °C. The solution was then allowed to cool to room temperature and stored in an amber bottle until further use [14].

#### 2.2.2. PVP-AgNPs

PVP-AgNPs were synthesized using a reflux method. First, a solution of 10 mL of 0.1 M silver nitrate in 100 mL of ethanol was prepared, followed by the addition of 1.00 g of PVP as a reducing and stabilizing agent, maintaining a silver nitrate to PVP weight ratio of 1:10. To prepare the PVP solution, ethanol and PVP were placed in a round-bottom flask and heated to 90 °C with gentle stirring for 30 min. Silver nitrate was then added, and the mixture was refluxed with continuous stirring for 7 h. Formation of PVP-AgNPs was visually confirmed by the development of an amber color, characteristic of AgNP surface plasmon resonance [8].

#### 2.2.3. Physicochemical and Morphological Characterization of AgNPs

The reduction of silver nitrate and formation of AgNPs were monitored using a UV–Vis spectrophotometer (JASCO V-770 UV-VIS/NIR; Tokyo, Japan) within a wavelength range of 200–800 nm to identify the characteristic localized surface plasmon resonance (LSPR) peak. Synthesis conditions were optimized by preparing AgNPs using varying concentrations of the reducing agents [15].

The functional groups associated with GLU-AgNPs and PVP-AgNPs were identified by Fourier-transform infrared (FTIR) spectroscopy. Spectra were recorded in suspension at room temperature using a Bruker Alpha II spectrophotometer at a resolution of 4 cm^−1^ in transmission mode over a range of 4000–400 cm^−1^. (Bruker Optics GmbH & Co. KG, Ettlingen, Germany). Spectral data were processed and plotted using Origin Pro 2019.

Nanoparticle size and morphology were evaluated using TEM (JEOL JEM-2011; Tokyo, Japan) at an accelerating voltage of 120 kV. A 5 μL aliquot of each AgNP suspension was deposited onto carbon-coated copper grids and dried in a silica desiccator for 16 h. TEM images were acquired to confirm nanoparticle shape, size, and distribution. Size distribution histograms were constructed using ImageJ software 1.54k [16]. For each nanoparticle formulation, four images at 6–40 kx magnification were analyzed, and at least 15 nanoparticles were counted to estimate mean size and range. This procedure was carried out across four independent micrographs for the silver nanoparticles (AgNPs).

Furthermore, hydrodynamic diameter and polydispersity index (PDI) were measured by dynamic light scattering (DLS). PDI values ≤0.1 were considered highly monodisperse; 0.1–0.25 moderately monodisperse; and >0.3 polydisperse. Zeta potential and conductivity were assessed using the same nanoparticle analyzer (Litesizer 500, (Anton Paar, Graz, Austria) at a scattering angle of 173 °C and 25 °C [15,17]. These measurements provided insights into colloidal stability and surface physicochemical characteristics.

### 2.3. Cell Culture and Treatment Conditions

JURKAT cells were obtained from the American Type Culture Collection (ATCC; Manassas, VA, USA). JURKAT is an immortalized human T-lymphocyte cell line derived from the peripheral blood of a 14-year-old male with acute T-cell lymphoblastic leukemia. PBMCs were isolated from peripheral whole blood collected from a healthy 19-year-old donor using density-gradient centrifugation with Lymphoprep™ (Serumwerk Bernburg AG, Bernburg, Germany), following the manufacturer’s instructions. Blood was carefully layered over Lymphoprep™ at a 3:1 ratio (blood:Lymphoprep) and centrifuged at 1800 rpm for 30 min at room temperature without brake. The PBMC layer was collected and washed three times with PBS (300× *g*, 5 min). Viability was determined by trypan blue exclusion (>95%). JURKAT cells were maintained by passaging every 2–3 days [18].

The study was conducted in accordance with the principles of the Declaration of Helsinki and approved by the Biosafety and Ethics Committees of the Centro Universitario de Ciencias de la Salud, Universidad de Guadalajara (protocol CI-06422).

Cell cultures were maintained in RPMI-1640 medium (Thermo Fisher, Waltham, MA, USA) supplemented with 10% fetal bovine serum (FBS) and 1% penicillin-streptomycin at 37 °C in a humidified atmosphere of 5% CO_2_. Cells were seeded at 1 × 10^4^ cells/mL in 96-well plates and exposed to AgNPs at concentrations of 6, 3, 1.5, 0.75, and 0.37 µg/mL for 24 h. Untreated cells served as negative controls, and etoposide-treated cells served as positive controls. All experiments were performed in triplicate.

### 2.4. Cell Viability Assay, IC_50_, and Selectivity Index

Cell viability in JURKAT cells was evaluated using the trypan blue exclusion assay. Cells were treated with GLU-AgNPs or PVP-AgNPs at concentrations of 0.37, 0.75, 1.5, 3, and 6 µg/mL for 24 h. After incubation, cells were stained with 0.4% trypan blue, and viable versus non-viable cells were counted using a Neubauer chamber under an optical microscope [19]. The same procedure was applied to PBMCs for comparative cytotoxicity assessment.

IC_50_ values were determined from dose–response curves generated from triplicate experiments. Concentrations were log-transformed for curve fitting, and IC_50_ values are reported in µg/mL. The selectivity index (SI) was calculated as SI = IC_50_(PBMC)/IC_50_(Leukemia), with SI > 1 indicating selective cytotoxicity toward leukemic cells.

### 2.5. Cytotoxicity Assay

The cytotoxicity of each AgNPs was evaluated using the MTT assay. AgNPs were added to the wells at concentrations of 0.37, 0.75, 1.5, 3, and 6 µg/mL, and the cells were incubated for 24 h. All experiments were performed in triplicate and included an internal control (without cells) for each experiment. This internal control contained only supplemented medium, the corresponding volume of each nanoparticle concentrations, and MTT. The absorbance values obtained from these internal controls were used exclusively to verify potential background interference and were not included in the final viability analysis.

An MTT stock solution was prepared by dissolving 5 mg of MTT in 1 mL of phosphate-buffered saline (PBS). A volume of 10 µL of this solution was added to each well, and plate was incubated for 4 h to allow the formation of formazan crystals. Subsequently, 100 µL of extraction buffer—prepared using sodium dodecyl sulfate (cat. no. 28312, Thermo Fisher Scientific^TM^, Waltham, MA, USA) and dimethylformamide (cat. no. 227056 Sigma-Aldrich^TM^, St. Louis, MO, USA)—was added to dissolve the formazan crystals. The plate was then incubated for an additional 18 h. Absorbance was measured at 570 nm using a BioTek^®^ 800™ spectrophotometer. Because residual glucose present in GLU-AgNPs samples may interfere with absorbance, a background correction at 490 nm was performed specifically for those samples to ensure accuracy.

### 2.6. Statistical Analysis

Dose–response curves were generated, and the half-maximal inhibitory concentration (IC_50_) values were determined by nonlinear regression analysis using a four-parameter logistic (4PL) model (log[inhibitor] vs. response—variable slope) in GraphPad Prism software v8. Concentrations were analyzed on a logarithmic scale to account for the wide range of tested values. Data normality was assessed using the Shapiro–Wilk test.

Quantitative variables—including IC_50_ values and the physicochemical characterization data of AgNPs—were expressed as mean ± standard deviation (SD). Comparisons between two groups were conducted using the unpaired Student *t*-test, whereas comparisons among three or more groups were performed using one-way ANOVA with Tukey’s post hoc test. When data did not meet parametric test assumptions, non-parametric tests (Mann–Whitney U or Kruskal–Wallis) were applied.

Qualitative variables were expressed as frequencies and percentages, and comparisons were performed using the Chi-square (χ^2^) test or Fisher’s exact test when expected cell counts were <5. All analyses were performed using GraphPad Prism v8 or R version 4.4.1 (build 24.12.1+563), with statistical significance set at *p* < 0.05 (two-tailed unless otherwise specified). All experiments were performed in triplicate to ensure reproducibility.

## 3. Results

### 3.1. Characterization of Silver Nanoparticles

**Figure 2:**
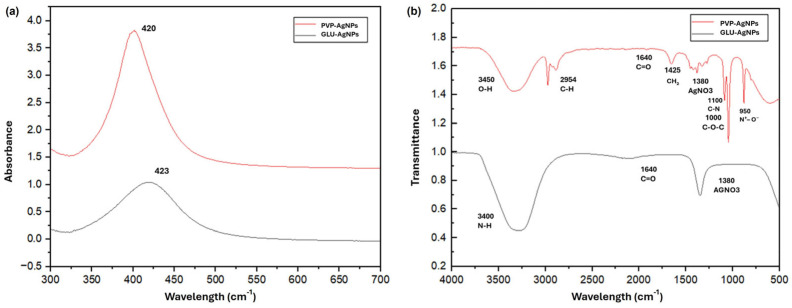
Physicochemical characterization of AgNPs synthesized with glucose (GLU) or polyvinylpyrrolidone (PVP); (**a**) UV-Vis spectra of GLU-AgNPs and PVP-AgNPs; (**b**) FTIR spectra of GLU and PVP-AgNPs.

According to the UV-Vis absorption spectra (Figure 2a), the synthesized AgNPs exhibited surface plasmon resonance (SPR) peaks at 423 nm for GLU-AgNPs and 420 nm for PVP-AgNPs, confirming nanoparticle formation. The slightly higher absorbance intensity observed for PVP-AgNPs may reflect differences in particle concentration, size distribution, or aggregation state compared with GLU-AgNPs.

FTIR analysis (Figure 2b) revealed distinct surface functional groups associated with each capping agent. Both nanoparticle types showed a band at ~1380 cm^−1^, corresponding to residual nitrate (NO_3_^−^) from unreacted silver nitrate rather than AgNO_3_ as a functional moiety. In GLU-AgNPs, characteristic bands were observed at 3417 cm^−1^ (O–H stretching from hydroxyl groups of glucose and/or gelatin), 1640 cm^−1^ (C=O stretching, possibly from carboxylate or amide I when gelatin is present), and 1050 cm^−1^ (C–O stretching typical of carbohydrates). These oxygen-rich groups likely contribute to nanoparticle stabilization through hydrogen bonding and steric effects.

For PVP-AgNPs, the spectrum displayed bands at 3448 cm^−1^ (O–H, adsorbed water), 2954 cm^−1^ (aliphatic C–H), 1640 cm^−1^ (C=O of the pyrrolidone ring), 1425 cm^−1^ (CH_2_ bending/C–N), 1000 cm^−1^ (C–O–C), along with features at 3050 cm^−1^ (C=C–H alkene) and 950 cm^−1^ (N^+^–O^−^, amine oxide), consistent with PVP’s structure and partial oxidation during synthesis. These nitrogen- and oxygen-containing groups enhance colloidal stability via steric hindrance and surface coordination.

Regarding colloidal properties (Table 1), PVP-AgNPs exhibited a hydrodynamic diameter of 30.0 ± 1.8 nm (PDI = 0.280), with particle sizes ranging from 6.3 to 60.7 nm. GLU-AgNPs showed a smaller hydrodynamic diameter of 22.4 ± 1.2 nm (PDI = 0.232), with sizes ranging from 10.0 to 47.6 nm. Zeta potential values were −10.01 ± 1.8 mV for PVP-AgNPs and −3.5 ± 1.2 mV for GLU-AgNPs, with conductivities of 0.163 mS/cm and 0.179 mS/cm, respectively.

**Figure 3:**
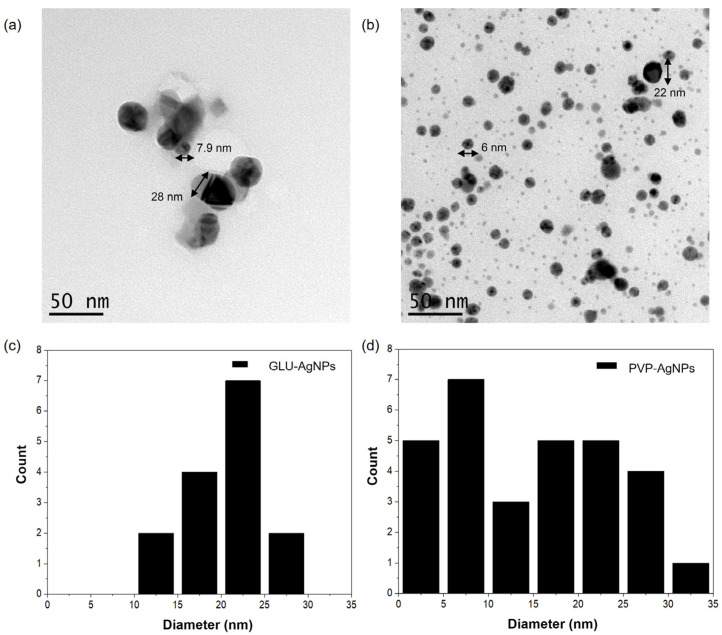
Transmission electron microscopy (TEM) images of (**a**) GLU-AgNPs and (**b**) PVP-AgNPs (scale bar: 0.2 µm), and size distribution histograms of (**c**) GLU-AgNPs and (**d**) PVP-AgNPs.

TEM analysis revealed clear morphological differences between the nanoparticle types (Figure 3). GLU-AgNPs displayed particle sizes ranging from 7.9 to 28 nm, with a mean diameter of 21.1 nm, exhibiting slight irregularities in shape and rougher surface texture. In contrast, PVP-AgNPs were predominantly spherical and demonstrated two distinct size populations ranging from 6 to 22 nm, with an average of 15.3 nm. These particles were well dispersed, exhibited smooth surfaces, and showed minimal aggregation. The size distribution histogram for PVP-AgNPs displayed two prominent peaks at 6 nm and 22 nm, consistent with the observed bimodal distribution.

### 3.2. Cell Viability and IC_50_

**Figure 4:**
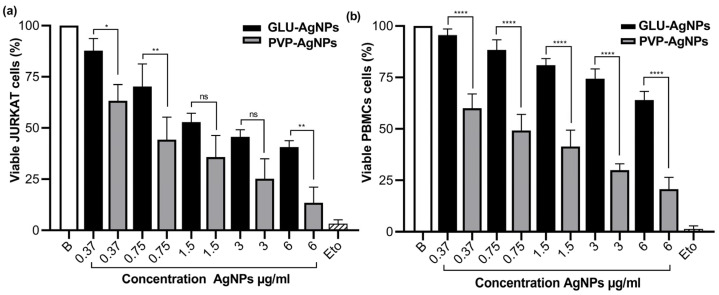
Percentage of viability after exposure to different concentrations of GLU-AgNPs (black bars) and PVP-AgNPs (gray bars); in (**a**) JURKAT and (**b**) PBMCs. Values represent mean ± SD from three independent experiments. B: blank, Eto: etoposide. Statistical analysis was performed using one-way ANOVA with Tukey’s post hoc test (* *p* < 0.05, ** *p* < 0.01, **** *p* < 0.0001; ns = not significant).

A dose-dependent decrease in cell viability was observed for both GLU-AgNPs and PVP-AgNPs in JURKAT cells and PBMCs (Figure 4). In JURKAT cells, PVP-AgNPs induced a significantly sharper reduction in viability than GLU-AgNPs. This difference was most pronounced at the highest concentrations tested: at 6 µg/mL, PVP-AgNPs reduced viability to 15%, whereas GLU-AgNPs maintained viability at approximately 40% (*p* < 0.01) (Figure 4a).

In PBMCs, GLU-AgNPs preserved viability above 60% at 6 µg/mL, whereas PVP-AgNPs reduced viability to approximately 20% at the same concentration (*p* < 0.001) (Figure 4b). These findings indicate that GLU-AgNPs exhibit lower cytotoxicity toward healthy immune cells than PVP-AgNPs.

**Figure 5:**
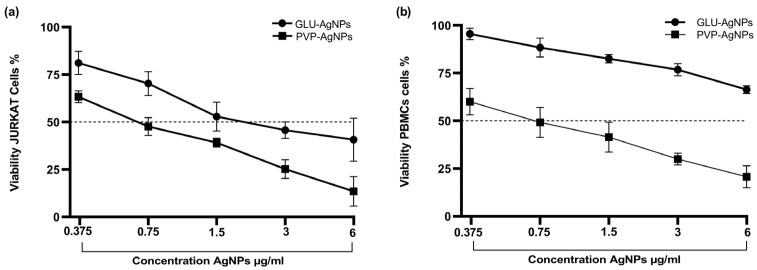
Dose–response curves for IC_50_ determination of GLU-AgNPs and PVP-AgNPs in (**a**) JURKAT cells and (**b**) PBMCs after 24 h of exposure at 0.37, 0.75, 1.5, 3.0, and 6.0 µg/mL. Data represent mean ± SD of three independent experiments. The dotted line represents the IC50.

Dose–response curves were used to determine IC_50_ values (Figure 5). In JURKAT cells, PVP-AgNPs showed a lower IC_50_ (0.85 µg/mL) compared with GLU-AgNPs (2.83 µg/mL), indicating greater cytotoxic potency. In PBMCs, GLU-AgNPs demonstrated an IC_50_ of 6.9 µg/mL, whereas PVP-AgNPs demonstrated a markedly lower IC_50_ (0.30 µg/mL), reflecting substantially higher toxicity toward healthy cells.

Selectivity was assessed using the Selectivity Index (SI), calculated as IC_50_ in PBMCs divided by IC_50_ in JURKAT cells (Table 2). GLU-AgNPs exhibited an SI of 2.44, indicating selective toxicity toward leukemic cells while sparing PBMCs. Conversely, PVP-AgNPs presented an SI of 0.36, demonstrating non-selective cytotoxicity and disproportionately greater toxicity toward normal immune cells.

### 3.3. Cytotoxicity in JURKAT and PBMCs Stimulated with GLU-AgNPs and PVP-AgNPs

**Figure 6:**
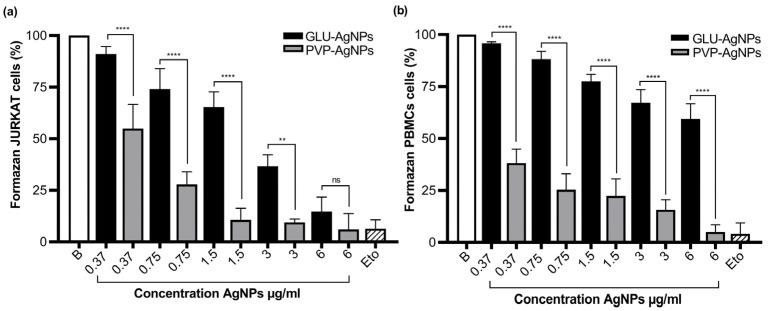
Formazan production (%) in JURKAT and PBMCs exposed to GLU-AgNPs (black bars) or PVP-AgNPs (gray bars); (**a**) JURKAT and (**b**) PBMCs. Values represent mean ± SD from three independent experiments. B: blank, Eto: etoposide. Statistical comparisons were conducted using one-way ANOVA with Tukey’s post hoc test (** *p* < 0.01, **** *p* < 0.0001; ns = not significant).

Cytotoxicity was dose-dependent in both JURKAT and PBMCs, as determined by metabolic activity via the MTT assay. In JURKAT cells (Figure 6a), metabolic activity decreased from 91.1% to 14.7% across concentrations of 0.37 µg/mL to 6 µg/mL of GLU-AgNPs, indicating substantial impairment of mitochondrial function in leukemic cells. This decline is consistent with the previously calculated IC_50_ (6.9 µg/mL), confirming that GLU-AgNPs require comparatively higher concentrations to induce half-maximal cytotoxicity, reflecting moderated cytotoxic potency.

In PBMCs (Figure 6b) GLU-AgNPs generated a milder response, with metabolic activity decreasing from 95.9% to 59.4% across the same concentration range. The IC_50_ remained above the highest concentration tested, suggesting reduced sensitivity and indicating selectivity cytotoxicity toward malignant cells.

By contrast, PVP-AgNPs exhibited strong and non-selective cytotoxicity. In JURKAT cells, viability dropped sharply from 54.9% to 6.0%, while PBMC survival decreased from 38.2% to 5.0%. These findings align with the low IC_50_ off PVP-AgNPs (0.30 µg/mL), demonstrating high cytotoxic potency without selectivity, as similar toxicity levels were observed in both malignant and healthy cells.

## 4. Discussion

In recent years, the use of AgNPs as novel therapeutic and immunomodulatory agents has gained considerable attention, particularly those synthesized using naturally occurring, biocompatible, and environmentally friendly reducing and capping agents [20]. For instance, AgNPs synthesized using *Achillea millefolium* extract demonstrated selective cytotoxicity against lymphoblastic leukemia cells (MOLT-4) [10]. In the context of leukemia, AgNPs have shown promising activity in inducing cell death in both myeloid (e.g., HL-60) and lymphoid (e.g., JURKAT) leukemia cell lines. Green-synthesized AgNPs from *Ziziphora tenuior* extract, for example, have been reported to trigger caspase-dependent apoptosis in HL-60 cells through ROS generation [21].

In our study, AgNPs were successfully synthesized via chemical reduction using glucose (GLU) as a reducing agent under multiple experimental conditions. UV-Vis spectroscopy revealed LSPR peaks at 423 nm for GLU-AgNPs and 420 nm for PVP-AgNPs, both within the expected range for spherical silver nanoparticles (400–430 nm) [7,22,23]. The slight red shift observed in GLU-AgNPs, together with their narrower absorption band compared with that of PVP-AgNPs, suggests differences in particle size distribution, shape homogeneity, and surface capping. The broader SPR band of PVP-AgNPs indicates greater heterogeneity in size and morphology, which is consistent with the TEM observations. These findings are aligned with established nanoplasmonic principles, where in the full width at half maximum (FWHM) of the SPR peak correlates strongly with nanoparticle polydispersity and surface roughness [21]. Our results also agree with those by Aguilar-Méndez et al. (2010) [14], who described LSPR peaks in the 420–423 nm range for similarly synthesized AgNPs [18].

FTIR analysis further confirmed the presence of distinct functional groups associated with each capping agent, emphasizing that the nature of the reducing and stabilizing molecules directly shapes the surface chemistry of AgNPs. In GLU-AgNPs, characteristic absorption bands at 3417 cm^−1^ (O–H and N–H stretching) and 1648 cm^−1^ (C=O in amide I) indicated strong interactions between GLU and the nanoparticle surface, suggesting enhanced biocompatibility [14]. In contrast, PVP-AgNPs exhibited characteristic peaks at 1654 cm^−1^ (C=O stretching of the pyrrolidone group), 2954 cm^−1^ (C-H stretching), and 3448 cm^−1^ (O-H stretching), confirming the stabilization of nanoparticles by PVP [7,21].

Zeta potential analysis indicated values of −2.5 mV for GLU-AgNPs and −10.01 mV for PVP-AgNPs, suggesting a comparatively lower electrostatic stability for GLU-AgNPs, although both remained stable in suspension [21]. Nevertheless, their low zeta potential suggests that GLU-AgNPs may agglomerate more easily than PVP-AgNPs. According to classical colloidal stability theory, values exceeding ± 30 mV are typically associated with strong electrostatic stabilization; however, steric and entropic contributions—particularly from capping agents such as gelatin—may counterbalance low zeta potentials [21,22]. Indeed, the higher stability inferred for GLU-reduced nanostructures, reflected by their lower PDI, may be attributed to the ability of gelatin to form a steric protective layer that prevents aggregation. DLS and TEM analyses confirmed that GLU-AgNPs presented predominantly spherical morphology and a more uniform size distribution ranging between 6 and 28 nm [14]. These structural characteristics are closely associated with their subsequent biological behavior, as reflected in the cell viability assays [7,23].

The influence of the reducing agent on cell viability was evident in the differential behavior between GLU- and PVP-based nanoparticles. GLU-AgNPs induced a gradual, concentration-dependent decrease in viability, whereas PVP-AgNPs produced a more abrupt decline, particularly in JURKAT cells. This finding supports the notion that nanoparticle-cell interactions are strongly affected by the type of reducing and capping agents [7,23].

Our results confirm that PVP-AgNPs exhibit significantly higher cytotoxicity and lower selectivity compared with GLU-AgNPs. This differential behavior is quantitatively represented by the selectivity index (SI), calculated as the ratio of IC_50_ in PBMCs to IC_50_ in JURKAT cells. GLU-AgNPs exhibited an SI of 2.439, indicating that a more than twofold higher concentration is required to induce toxicity in healthy cells—consistent with a favorable therapeutic window [7,19]. In contrast, PVP-AgNPs produced an SI of 0.361, demonstrating inverted selectivity and greater toxicity toward normal immune cells, limiting their potential application in targeted anticancer therapy [13,23].

The observed selectivity may be explained by intrinsic metabolic differences between malignant and healthy immune cells. Leukemic cells, such as JURKAT, exhibit elevated glucose uptake due to overexpression of glucose transporters (e.g., GLUT1) a hallmark of cancer metabolism. This metabolic phenotype may promote preferential internalization of GLU-AgNPs [19]. Conversely, PBMCs, being quiescent primary cells, display lower metabolic activity and endocytic activity, which may limit nanoparticle uptake and cytotoxicity. By contrast, the synthetic PVP corona may facilitate non-specific cellular interactions and uptake, contributing to indiscriminate cytotoxicity in both malignant and non-malignant cells [24,25,26].

Previous studies have demonstrated that nanoparticle surface functionalization can be strategically modulated to tune the therapeutic profile of AgNPs, enhancing either biocompatibility or cytotoxic potency depending on the clinical objective [7,27]. Our findings position GLU-AgNPs as a promising candidate for safer anticancer applications in which the preservation of immune function is essential.

Taken together, these results demonstrate that the reducing agent used during AgNP synthesis substantially influences their biological activity. Whereas PVP-AgNPs exhibited potent but non-selective cytotoxicity, GLU-AgNPs displayed a more favorable safety profile, with minimal effects on healthy PBMCs [28]. Although these characteristics support the potential use of GLU-AgNPs in clinical settings, further in vivo studies are required to fully assess their therapeutic safety and efficacy. Additionally, future research should explore their long-term biological effects, their interaction with conventional anti-leukemic drugs, and their performance in three-dimensional lymphoid tissue models [29,30,31].

## 5. Conclusions

This study demonstrates that the reducing agent used during the synthesis of AgNPs significantly influences their physicochemical properties and their interactions with leukemic and healthy cells. GLU-AgNPs exhibited higher colloidal stability and a less pronounced cytotoxic profile, resulting in higher cell viability and a favorable selectivity index (SI = 2.439) toward leukemic cells. In contrast, PVP-AgNPs showed a slightly higher polydispersity (i.e., a bimodal size distribution profile) and a more negative zeta potential, which likely facilitated their cellular uptake, resulting in a stronger cytotoxic effect on leukemic cells and lower cell viability compared to GLU-AgNPs. These findings confirm that the chemical nature of the reducing agent and the capping layer formed during synthesis not only affect the physicochemical characteristics and colloidal behavior of AgNPs but also modulate the biological responses of exposed cells.

Overall, this work underscores the importance of nanoparticle design as a strategic tool to tailor their biological performance. The choice of reducing agent emerges as a key factor in the development of oncological nanomedicine, although further in vivo studies and mechanistic investigations are needed to fully assess its implications.

## Figures and Tables

**Table 1:** Hydrodynamic diameter, polydispersity index (PDI), and zeta potential of GLU-AgNPs and PVP-AgNPs.

Property	GLU-AgNPs	PVP-AgNPs
Hydrodynamic diameter (nm)	22.4 ± 1.20	30 ± 1.8
Polydispersity index (PDI)	0.232	0.280
Zeta potential (mV)	−3.50	−10.01

**Table 2:** IC_50_ values and Selectivity Index (SI) for GLU-AgNPs and PVP-AgNPs in JURKAT and PBMCs.

Cell Type	AgNPs	IC_50_ (μg/mL)	SI
JURKAT	GLU-AgNPs	2.830	2.439
	PVP-AgNPs	0.309	
PBMCs	GLU-AgNPs	6.910	0.361
	PVP-AgNPs	0.854	

## Data Availability

The data presented in this study are available within the article.

## Abbreviations

AgNPs	Silver Nanoparticles
GLU	Glucose
PVP	Polyvinylpyrrolidone
PBMCs	Peripheral Blood Mononuclear Cells
SPR	Surface Plasmon Resonance
FTIR	Fourier-Transform Infrared Spectroscopy
TEM	Transmission Electron Microscopy
DLS	Dynamic Light Scattering
MTT	3-(4,5-Dimethylthiazol-2-yl)-2,5-diphenyltetrazolium bromide
IC_50_	Half-Maximal Inhibitory Concentration
SI	Selectivity Index
ANOVA	Analysis of Variance
SD	Standard Deviation
CUCS	Centro Universitario de Ciencias de la Salud
INICIA	Instituto de Investigación en Cáncer en la Infancia y Adolescencia
IICB	Instituto de Investigación en Ciencias Biomédicas
